# Special issue devoted to the IAPC-10 Meeting: Joint World Conferences on Physico-Chemical Methods in Drug Discovery and Development and on ADMET and DMPK

**DOI:** 10.5599/admet.2541

**Published:** 2023-10-31

**Authors:** Tatjana Verbić, Zoran Mandić

**Affiliations:** 1 University of Belgrade – Faculty of Chemistry, Studentski trg 12-16, 11158 Belgrade, Serbia E-mail: *tatjanad@chem.bg.ac.rs*; Tel.: +381 63 255356; 2 University of Zagreb, Faculty of Chemical Engineering and Technology, HR-10000, Zagreb, Croatia E-mail: *zmandic@fkit.hr*; Tel.: +385 1 4597164

The present issue of ADMET and DMPK is dedicated to the IAPC-10 Meeting, which was organized as a joint event consisting of 10^th^ World Conference on Physico-Chemical Methods in Drug Discovery and Development and 6^th^ World Conference on ADMET and DMPK. The meeting took place in the University of Belgrade Rectorate building, Belgrade, Republic of Serbia, September 4-6, 2023. IAPC meetings are organized as annual events in alternating European and East Asia locations. The topics covered the most advanced directions and new achievements in physico-chemical methods, which underlie almost all instrumental techniques used in the research processes in drug discovery and pharmaceutical development. Experimental determination of ADMET properties through the i*n vitro* and *in vivo* assays was discussed as well as modern theoretical methods for computer-aided drug design. Ten sessions were organized, among which two special sessions: 1) Special Session on Solubility of Multi-Component Solids (Salts and Cocrystals), organized and co-chaired by Kiyohiko Sugano and Alex Avdeef, and 2) Special Session on the ADME properties and toxicity prediction by HPLC, organized and chaired by Klara Valko.

Almost 100 delegates actively participated in the IAPC-10 Meeting. Most of them presented their work orally or through a poster presentation, resulting in a diverse and fully-filled three-day program. A selection of papers (five original scientific papers and one short communication) giving a typical cross-section of the conference workings is published in this special issue.

Diego Garcia Jimenez *et al.* report the full physicochemical characterization of three PROTACs in clinical trials; the paper focuses on the molecular properties essential to drive oral bioavailability within the beyond rule of five (bRo5) drug framework. Susana Amézqueta *et al.* discuss the micellization parameters of surfactants and describe different probes and data treatment models to test and set up an analytical method based on fluorescence measurements to determine the critical micelle concentration (CMC) and micellization range (a*C*) of biosurfactants, with sodium taurocholate (NaTc) as a case study. Chrysanthos Stergiopoulos *et al.* have measured the chromatographic retention of the 13 UV filters on an immobilized artificial membrane (IAM) stationary phase to assess phospholipid binding to use these measurements, combined with previously established predictive models developed for pharmaceutical compounds, to predict the acute aquatic toxicity of organic UV filters used in cosmetic formulations.

Shan Lu *et al.* discuss physiologically based pharmacokinetics (PBPK) modelling, coupled with pharmacodynamics (PD) simulations, to explore possible factors determining drug nanocrystals' biodistribution and treatment efficacy. Slavica Oljačić *et al.* described the application of Multiple Linear Regression and Partial Least Squares Regression for the quantitative structure-retention relationship (QSRR) and quantitative structure-mobility relationship (QSMR) to model and select the most important molecular parameters describing the chromatographic and electrophoretic behaviour of imidazoline and alpha-adrenergic receptors ligands under different acid-base conditions.

Michele Domenico Spampinato *et al.* wrote a short communication on *ex vivo* propofol permeation across nasal mucosa as a proof-of-concept study for outpatient light sedation via nasal route; presented results highlight the key role of formulation and the need for innovation in solubility and transmucosal transport enhancement techniques to optimize drug delivery and therapeutic efficacy.

We wish to thank all the authors of this special issue for their high-quality papers and all the referees for their time, expertise, and efforts put into critical evaluations of the submitted papers. We also truly hope that the readers will enjoy the published manuscripts and find them interesting, useful and beneficial for their future work.

**Figure:**
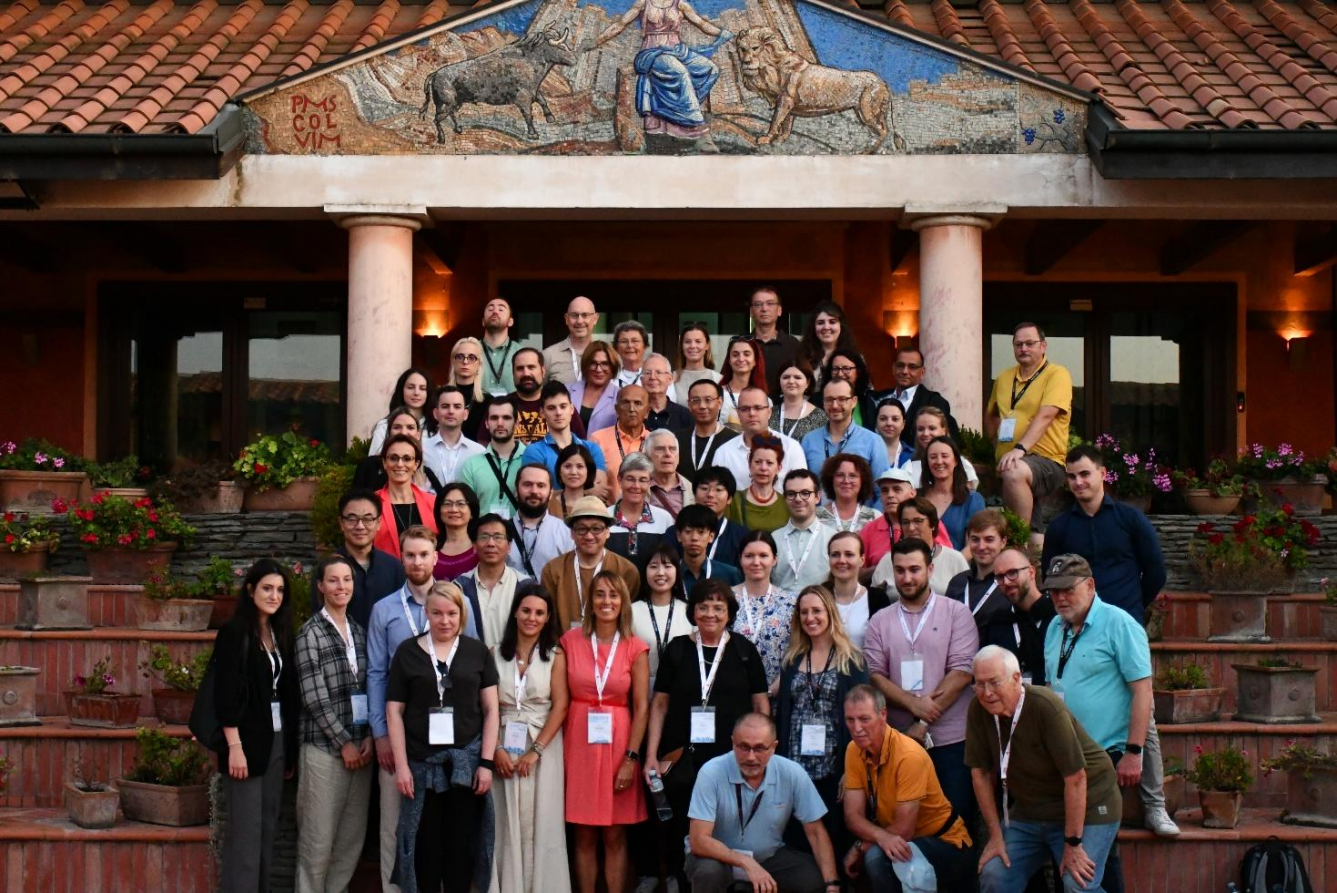
Conference participants photo taken during the excursion to Viminacium – Roman City and Legionary Fort.

